# Dynamic mRNA and miRNA expression analysis in response to intermuscular bone development of blunt snout bream (*Megalobrama amblycephala*)

**DOI:** 10.1038/srep31050

**Published:** 2016-08-03

**Authors:** Shi-Ming Wan, Shao-Kui Yi, Jia Zhong, Chun-Hong Nie, Ning-Nan Guan, Wei-Zhuo Zhang, Ze-Xia Gao

**Affiliations:** 1 College of Fisheries, Key Lab of Agricultural Animal Genetics, Breeding and Reproduction of Ministry of Education/Key Lab of Freshwater Animal Breeding, Ministry of Agriculture, Huazhong Agricultural University, Wuhan, Hubei 430070, China; 2 Freshwater Aquaculture Collaborative Innovation Center of Hubei Province, Wuhan 430070, China; 3 Hubei Provincial Engineering Laboratory for Pond Aquaculture, Wuhan 430070, China

## Abstract

Intermuscular bone (IB), which occurs only in the myosepta of lower teleosts, is attracting more attention because they are difficult to remove and make the fish unpleasant to eat. By gaining a better understanding of the genetic regulation of IB development, an integrated analysis of miRNAs and mRNAs expression profiling was performed on *Megalobrama amblycephala*. Four key development stages were selected for transcriptome and small RNA sequencing. A number of significantly differentially expressed miRNAs/genes associated with bone formation and differentiation were identified and the functional characteristics of these miRNAs/genes were revealed by GO function and KEGG pathway analysis. These were involved in TGF-β, ERK and osteoclast differentiation pathways known in the literature to affect bone formation and differentiation. MiRNA-mRNA interaction pairs were detected from comparison of expression between different stages. The function annotation results also showed that many miRNA-mRNA interaction pairs were likely to be involved in regulating bone development and differentiation. A negative regulation effect of two miRNAs was verified through dual luciferase reporter assay. As a unique public resource for gene expression and regulation during the IB development, this study is expected to provide forwards ideas and resources for further biological researches to understand the IBs’ development.

It is well known that most of the freshwater aquaculture fishes around the world, especially Cyprinidae species, possessed a certain amount of intermuscular bones (IBs), which are hard-boned spicules located in the muscle tissue on both sides of the vertebrae^1^. The IBs develop directly from mesenchymal condensation and consist of membranous ossifications of connective tissue in the muscular septum. IBs differ from other axial skeletons, such as the vertebral column, which develops from a mesenchymal cell population derived from the ventral somite^2^. IBs reduce the edibleness and economic value of a fish species^3^. In view of the potential value in eliminating IBs, researchers began to study IBs in fish as early as the 1960s^4^. Interestingly, prior studies revealed the significant differences in the number of IBs among fish of different ploidy, indicating that genetic improvement methods could have a significant effect on decreasing the number of IBs^5^. But so far, almost all existing studies have only focused on the morphology of IBs in fish species^6^,^7^,^8^. Few in-depth studies have been carried out and little information has been obtained regarding the molecular mechanism for development of IBs. Only one previous study has revealed the molecular properties of IBs through microRNA (miRNA) transcriptome analysis^9^.

High-throughput sequencing technologies are being widely used to investigate the complete repertoire of expressed RNA transcripts in specific tissues or cells at specific stages of development, and this knowledge is helping improve our understanding of the molecule mechanism affecting expression^10^, regulatory^11^ and development^12^. In fish, independent transcriptome profiling (RNA-seq) of miRNAs or mRNAs has been used to identify gene and miRNA expression related to various physiological functions^13^,^14^,^15^,^16^. As miRNAs has been reported to regulate gene expression by promoting mRNA degradation and repressing translation, the combined analyses of mRNA with miRNA expression data has been conducted in plants and mammals to infer regulatory mechanisms associated with various physiological processes^17^,^18^,^19^. In fish species, the integrated analysis of mRNA and miRNA expression profiles has been performed on zebrafish (*Danio rerio*) for optic nerve regeneration and on darkbarbel catfish (*Pelteobagrus vachelli*) in response to hypoxia^20^,^21^.

Perazza *et al*.^22^ has reported that specimens of tambaqui (*Colossoma macropomum*) from one culture population lack IBs, even though this fish normally has IBs. Xu *et al*.^23^ has identified an intermuscular bone-deficient grass carp (*Ctenopharyngodon idellus*) mutant in an artificial gynogenetic group. Based on these two reports, it has been speculated that there maybe one or several key genes that regulate the development of IBs.

In the present study, we used one typical Cyprinidae species, blunt snout bream (*Megalobrama amblycephala*), for which the morphological characteristics, emergent periods and morphogenesis of IBs are known from our previous research^24^. IBs gradually appear during the growth and development of *M. amblycephala*. It emerged 20 days post hatching (dph) at a body length of 1.33 cm, first in the tail and then toward the head. When fry were 40 dph with the body length of 2.36 cm, all intermuscular bones had appeared. With the aim to generate the fundamental molecular resources across the different developmental stages from emerging to complete formation of IBs and try to find the putative key genes or miRNAs for regulating IBs’ development, the transcriptome property, mRNA and miRNA expression profiling were investigated in the four key stages related to IBs development of *M. amblycephala* in this study. A certain amount of bone-related genes, period-specific or period-differential expressed mRNAs and miRNAs were identified, and the related signaling pathways were uncovered through the comparisons of the four development stages. Meanwhile, some interaction networks and regulatory modes of mRNAs and miRNAs were also revealed based on the integrated analysis of miRNA and mRNA expression profiles.

## Materials and Methods

All animals and experiments were conducted in accordance with the “Guidelines for Experimental Animals” of the Ministry of Science and Technology (Beijing, China). The study was approved by the Institutional Animal Care and Use Ethics Committee of Huazhong Agricultural University. All efforts were made to minimize suffering. All experimental procedures involving fish were approved by the institution animal care and use committee of the Huazhong Agricultural University.

### Experimental animals and tissue collection

**Figure 1:**
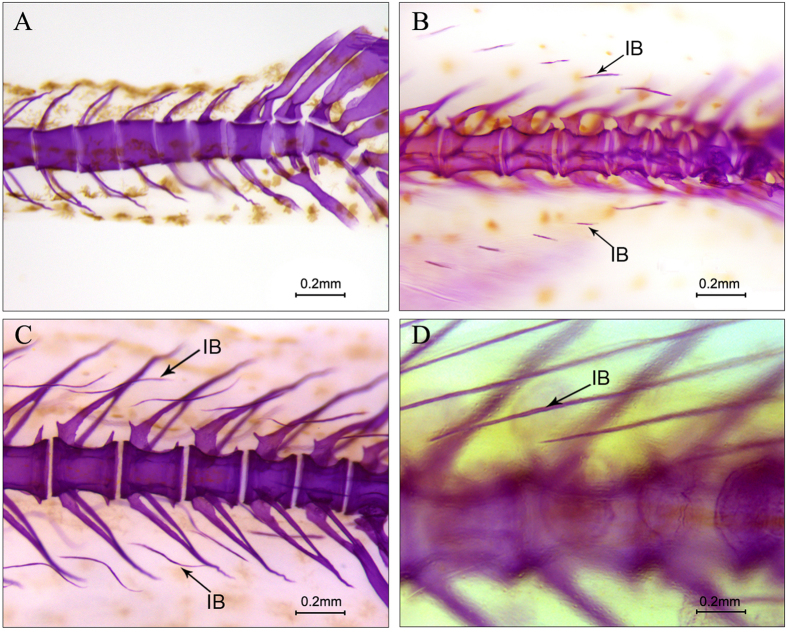
Development characteristics of IBs in the four key development stages of *M. amblycephala*. (**A**) Stage 1 (S1). Body Length ≈ 11 mm. 17 dph, the IBs haven’t emerged, but the fins and axial skeleton have developed completely, so the presence or absence of IBs does not affect the development of fins and axial skeleton; (**B**) Stage 2 (S2). Body Length ≈ 16 mm. 24 dph, a few IBs of small length have emerged in the tail; (**C**) Stage 3 (S3). Body Length ≈ 21 mm. 29 dph, more IBs of greater length gradually emerged in the tail; (**D**) Stage 4 (S4). Body Length ≈ 32 mm. 42 dph, all of the IBs in the tail have a mature morphology and length. IBs have also emerged throughout the body of the fish from the tail to the head.

**Figure 2:**
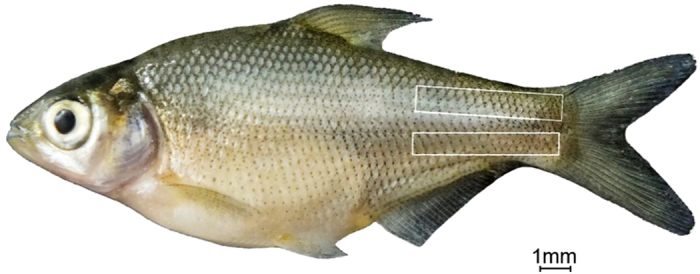
Sampling area for the study (rectangular area; Fish from S3).

Experimental fishes were selected from an artificially propagated population and grown in the recirculating aquaculture system after hatching. Before sampling, experimental animals were anaesthetized in well-aerated water containing the 100 mg/L concentration of Tricaine methanesulfonate (MS-222). Four key stages for the development of IBs were selected as done for our previous research^21^ and shown in Fig. 1. Muscle tissue containing IBs in the tail (Fig. 2), which were the initial area of intermuscular bone differentiation, were sampled under an anatomical lens. Details about sampling are described in Supplementary Fig. S1-1. The accuracy of sample collection was validated using Alizarin red and Alcian blue staining to ensure that there was no interference form fins, scales and other impurities (Supplementary Fig. S1-2). Composite samples of 6–10 individuals were collected to extract total RNA for every stage and each stage possessed two biological replicates. In addition, six months old fish was used to sample different tissues for gene expression analysis.

### Total RNA isolation and cDNA library construction

Total RNA was isolated from the samples at 4 stages and different tissues using RNAiso Plus Reagent (TaKaRa, China) according to the manufacturer’s protocol. RNA quality and quantity were measured using the NanoDrop 2000 (Thermo Scientific, USA) and Agilent 2100 Bioanalyzer (Agilent, USA). All the samples were standardized to 500 ng/μl. At each stage, two Digital Gene Expression Profiling libraries and one miRNA transcriptome library were constructed. Meanwhile, a reference transcriptome library was constructed by mixing equal volumes RNA from 4 stages. The schematic diagram for high throughput sequencing is shown in Supplementary Fig. S1-3. During the QC steps, Agilent 2100 Bioanaylzer and ABI StepOnePlus Real-Time PCR System were used to quantify and qualify the sample library preparations. Finally, the libraries were sequenced using an Illumina HiSeq™ 2000 platform (BGI, Shenzhen, China). All of the raw RNA-Seq data were submitted to the NCBI databases (http://trace.ncbi.nlm.nih.gov/Traces/sra/sra.cgi?view=run_browser) under accession number SRR2335137 for transcriptome data, SRR2338087 for DGE data and SRR2338872 for miRNA data.

### *De novo* assembly and functional annotation

All reads with low quality or shorter than 18 nt were eliminated. The 5′ or 3′ primer contaminants and polyA tails were removed. The clean sequencing reads were de novo assembled using the Trinity (http://trinityrnaseq.sourceforge.net/) with *Kmer* = 25. The TGI Clustering Tool (version 2.1) (http://sourceforge.net/projects/tgicl/) was used for further processing of sequence splicing and redundancy removal. BLASTx (version v2.2.26) (E-value cut-off 1e-5) protein database (NR, Swiss-Prot, KEGG and COG) alignment was performed. When results from different databases conflicted, a priority order of NR, Swiss-Prot (http://www.ebi.ac.uk/swissprot/), KEGG (http://www.genome.jp/kegg) and COG (http://www.ncbi.nlm.nih.gov/COG) was followed for annotation. When a unigene was found to be unaligned to the above databases, ESTScan software (http://estscan.sourceforge.net/) was introduced to decide its sequence direction. Furthermore, biological function annotation and classification were performed by mapping unigenes to each term of the Gene Ontology database (http://www.geneontology.org/), and pathways for biochemical and signal transduction were determined via a KEGG pathway analysis (http://www.kegg.jp/).

### Gene expression profiling of different development stages

After raw data filtering, clean reads of the 8 libraries were mapped to the reference transcriptome using SOAPaligner/soap2 (http://soap.genomics.org.cn/) to obtain unique mapped reads, allowing a maximum of two mismatches in the alignment. The number of unique-match reads was calculated and normalized to RPKM (reads per kb per million reads) for gene expression analysis. Comparison of unigene expression between different stages was performed using DESeq analysis^25^. Genes with log-fold difference (log2Ratio) ≥1 and false discovery rate (FDR < 0.001) were considered to be significantly differentially expressed.

### MiRNA sequencing and differential expression analysis

Four small RNA libraries were constructed and sequenced as previously described^15^. High-quality reads were blasted against the Rfam (ftp://sanger.ac.uk/pub/databases/Rfam/) database and the GenBank noncoding RNA database (http://blast.ncbi.nlm.nih.gov/) to annotate rRNA, tRNA, snRNA and other ncRNA sequences, and then aligned to exons and introns of mRNA to screen and remove degraded fragments. Selected sequences were also mapped to the reference transcriptome with a tolerance of one mismatch in the seed sequence to analyze their expression and distribution on the genome by SOAP. Conserved miRNAs were identified through a Blastn search against the miRNA database, miRBase20.0 (http://www.mirbase.org/ftp.shtml) using Mireap software (https://sourceforge.net/projects/mireap/).

MiRNAs with reads less than 100 were discarded, and miRNA expression levels were normalized by TPM (transcript per million) values (TPM = (miRNA total reads/total clean reads) × 10^6^). Comparisons between developmental stages were made to find significantly differentially expressed miRNAs (|log2(fold change)| > 1 and P-value ≤ 0.05). Subsequently, gene expression differences between developmental stages showing complementarity with corresponding miRNA expression values were selected and analyzed with Targetscan (http://www.targetscan.org/) and MiRanda (http://www.microrna.org) to predict the miRNA target. Furthermore, GO terms and KEGG enrichment analysis on differential expressed miRNAs was determined via a hypergeometric test with FDR ≤ 0.05. In addition, detection and interaction analysis of miRNA-mRNA pairs were performed based on the targets prediction, function annotation and negative regulation mechanism of mRNA and miRNA. The miRNA-mRNA interaction networks were displayed using visualizing maps.

### Quantitative PCR for miRNA and mRNA expression

To validate the sequencing data, 9 miRNAs and 6 mRNAs showing sustained significant increases or declines in expression over the four stages and 13 miRNA-mRNA interaction pairs identified from the pairwise comparison of S1 and S3 (S1 and S3 showing significant differentiation in the number and form of IBs; Fig. 1) were selected, and specific primers were used to quantify the miRNA and mRNA for each stage of development (Supplementary Table S1). After acquiring high quality total RNA, miRNAs and mRNAs were reverse transcribed using PrimeScript RT reagent Kit with gDNA Eraser (TaKaRa, Japan, RR047A). Quantitative real-time PCR analyses on the miRNAs and the mRNAs were performed using the SYBR Green PCR Master Mix (TaKaRa, Japan, RR820A) on a Roche LightCycler 480 System II (Roche, Mannheim, Germany) according to the manufacturer’s instructions. *M. amblycephala* 18s RNA and β-actin were used as internal controls for miRNA and mRNA RT qPCR, respectively. All of the real-time reactions were performed in triplicate and the relative expression levels were measured in terms of threshold cycle value (Ct) and were normalized using the equation 2^−ΔΔCt^, in which ΔCt = Ct_miRNA/mRNA_ − Ct_18s/β-actin_. Based on the annotation information of mRNA transcriptome data, we selected a number of bone-related genes to analyze their expression in different tissues (Supplementary Table S2), with the aim to find IB specific genes.

### Vector Construction and Dual Luciferase reporter assays

The 3′ UTR of *tgfbr1a* and *runx2a* containing a miR-133b-3p binding site and the 3′ UTR of *tgfbr1a* and *runx2b* containing a miR-206-3p binding site were amplified from genomic DNA by PCR with the primers shown in Supplementary Tables S1-4. PCR products were cloned into pmirGLO using the MSS I and Xho I restriction sites. Dual luciferase reporter experiments were performed in HeLa cell lines (Cell Collection Center for Freshwater Organisms, Huazhong Agricultural University). When the cells reached 60% to 70% confluence in 24-well plates, pmirGLO-3′ UTR (200 ng) was co-transfected with 100 nM negative control or a microRNA mimics (GenePharma, Shanghai, China) using 2 μL Fugene6 (Promega) according to the manufacturer’s instructions. The relative luciferase activity was measured 24 h after transfection by the Dual-Luciferase Reporter Assay System (Promega).

## Results and Discussion

### Assembly and annotation of reference transcriptome

To obtain a reference transcriptome for the IBs’ development in *M. amblycephala*, a RNA-Seq library was constructed using RNA from samples of four development stages. A total of 92,991,884 raw reads were generated through high-throughput sequencing. After quality control, approximately 7,882,445,160 nt of high-quality data with a Q20 percentage of 98.11% and GC percentage of 48.70% was available for analysis (Table 1). With the Trinity *de novo* assembler, a total of 127,712 contigs were generated, with an average length of 301 bp and an N50 of 437 bp. A total of 52,918 unigenes were further generated with an average length of 635 bp and an N50 of 865 bp (Table 1). The size distribution of these contigs and unigenes is shown in Supplementary Fig. S1-4 and 5. A total of 46,569 unigenes were successfully annotated through alignment to reference databases. In total, 33,354 (63.03%), 45,648 (86.26%), 29,753 (56.22%), 24,094 (45.53%) and 9,072 (17.14%) unigenes could be annotated by NR, NT, Swiss-Prot, KEGG and COG database, respectively, with 6,349 (12.00%) unigenes showed no homology to known sequences deposited in these databases (Table 1).

Based on the NCBI nr database, E-value distribution (Supplementary Fig. S1-6A) and homology percent of the unigenes was performed and mostly of them showed strong homology to available database sequences (Supplementary Fig. S1-6B). Meanwhile, 30,258 (90.72%) unigenes were annotated to 5 top-hit species (*Brachydanio rerio*, *Oreochromis niloticus*, *Oryzias latipe*, *Fugu rubripes*, *Tetraodon nigroviridis*), especially *D. rerio* (81.41%) (Supplementary Fig. S1-6C). A total of 22,222 *M. amblycephala* unigenes were classified into 3 gene ontology (GO) categories (cellular component, biological process and molecular function) (Supplementary Fig. S1-7) and 24,094 unigenes were mapped into 258 KEGG pathways. Metabolic, regulation of actin cytoskeleton, cancer and focal adhesion pathways dominated the KEGG functional analyses. In addition, some pathways related to osteocyte differentiation, such as the MAPK (mitogen-activated protein kinase), Wnt signaling pathway, osteoclast differentiation and TGF-β (transforming growth factor beta) signaling pathways, were highly represented in the results of the KEGG functional analyses (Supplementary Table S3).

Based on the annotation, hundreds of bone-related genes, such as FGFRs, SOXs, Runx2, TGFβs, BMPs, SMAD, Osteocalein, MMP, cathepsin K, Col1a1/2, Col2a1, ColXa1, IGFs and so on, were identified in the IB transcriptome (Supplementary Table S4). These genes have a well-recognized function in formation and differentiation of cartilage and bone^26^. For example, the combination of SOX5, SOX6, and SOX9 suggests that the signals necessary for induction of permanent cartilage are present in the transcriptome^27^. BMP signaling is known to be involved in fish in fin growth, scleroblast differentiation^28^, tissue calcification^29^, and in mammals BMP and Ihh/PTHrP signaling interact to coordinate chondrocyte proliferation and differentiation^30^. At least 30 collagens isoforms have been identified in bone and are important in skeletal development^31^. TGFβ has a role in osteoblast differentiation and bone formation^32^. The identification in the transcriptome of abundant bone-related genes supports that our present transcriptome is closely related to the bone, which justifies the enrichment of the transcriptome for IBs.

### Expression profiling of IBs development-dependent genes

In order to identify the functional genes in response to development of IBs, 8 DGE (digital gene expression profiling) libraries have been generated from 4 developmental stages with two biological replicates. The mapping results for the 8 libraries (4 developmental stages) are shown in the Table 2. The distribution of unique reads in the 8 DGE libraries was compared as an assay of gene coverage. Similar coverage was obtained for all 8 libraries. More than 34% of the unigene sequences in every library have a gene coverage of more than 70% (Supplementary Fig. S1-8), which is determined as the ratio of the base number in a gene covered by unique mapping reads to the total bases number of that gene. Sequencing saturation analysis showed the number of unique tags reached a plateau shortly after the amount of clean reads reached 20 million. Therefore, the 8 libraries fully represent the transcripts expressed in each developmental stage. Principal component analysis (PCA) showed that the biological replicates had very similar expression levels, suggesting good reproducibility of the method (Supplementary Fig. S1-9).

**Figure 3:**
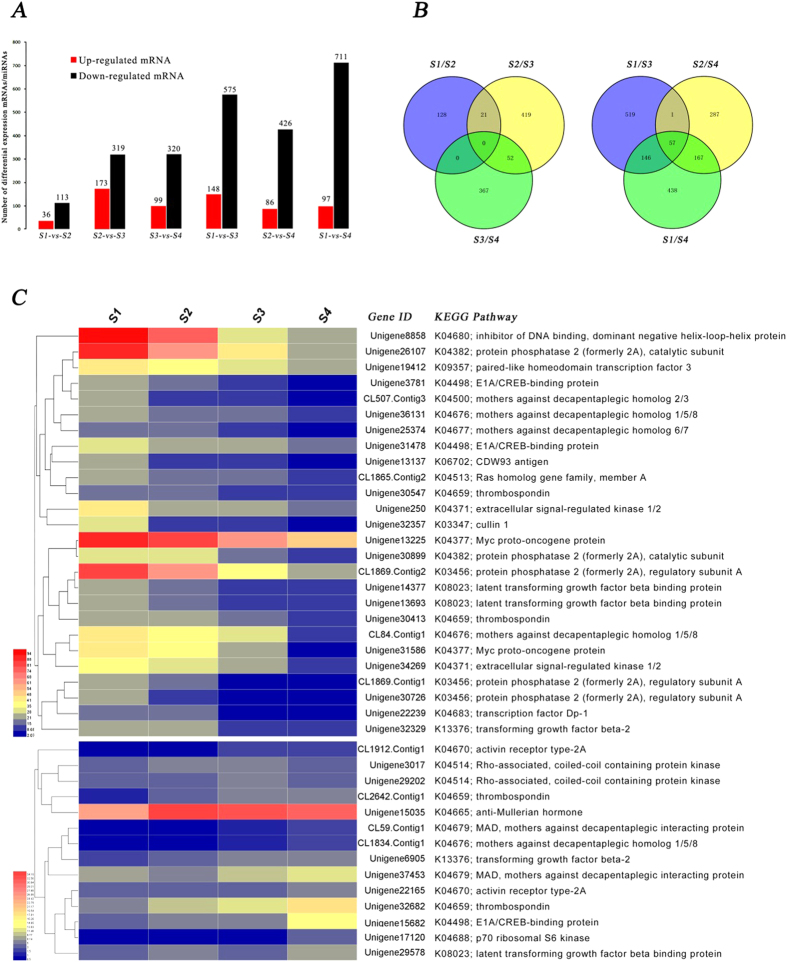
Differentially expressed profiles for mRNA. (**A**) The number of up/down-regulated DEGs in each comparison analysis group; (**B**) Venn diagram of differential expression between adjacent/nonadjacent pairwise comparisons; (**C**) Hierarchical cluster analysis for DEGs belonging to the TGF-β pathway and having a sustained decreased/increased expression levels from stages S1 to S4.

Comparison of adjacent developmental stages S1-vs-S2, S2-vs-S3 and S3-vs-S4 detected 36 up-regulated and 113 down-regulated genes, 173 up-regulated and 319 down-regulated genes, 99 up-regulated and 320 down-regulated genes, respectively (Fig. 3A, Supplementary Table S5). Pairwise comparisons between nonadjacent developmental stages S1-vs-S3, S2-vs-S4 and S1-vs-S4 detected 148 up-regulated and 575 down-regulated genes, 86 up-regulated and 426 down-regulated genes and 97 up-regulated and 711 down-regulated genes, respectively (Fig. 3A). Venn diagrams displayed no overlapping DEGs for the three adjacent pairwise comparisons, while 57 overlapping DEGs were identified with nonadjacent pairwise developmental stage comparisons (Fig. 3B). These results indicated that comparisons of nonadjacent developmental stages can obtain more key DEGs, which may play an important role during long-term development of IBs.

The analysis of specific expression genes (SEGs) found 59, 239, 569 and 470 development-dependent specific expression genes in the S1, S2, S3 and S4 stages, respectively (Supplementary Table S6). The number of SEGs increased with the development of IBs, which showed that more unigenes expressed and functioned with the appearance and development of IBs. The identification of SEGs related to development stages may provide key genomic information to explore the mechanism of IB development.

### Functional analysis of DEGs and SEGs during the four developmental stages

GO categorization of DEGs in the six pairwise comparisons were all significantly enriched in cellular process, cell, cell part, binding and single-organism process (Supplementary Table S5), indicating the similarity in the major processes that respond to different developmental stages of IBs. Through aligning to the KEGG database, which categorizes gene functions with emphasis on biochemical pathways, a total of 13, 92, 52, 184, 121 and 184 DEGs involved in 12, 35, 31, 50, 55 and 52 pathways were predicted in the pairwise comparison of S1-vs-S2, S2-vs-S3, S3-vs-S4, S1-vs-S3, S2-vs-S4 and S1-vs-S4 (Supplementary Table S7), respectively. The metabolic pathway containing the most DEGs was ko01100, which performs a variety of anabolic and catabolic tasks, affecting energy conversion, macromolecular compounds synthesis and so on^33^. Other pathways with an abundance of DEGs included those involved with the biosynthesis of secondary metabolites (ko01110), protein processing in endoplasmic reticulum (ko04141) and ribonucleotide metabolism (ko00240, ko00230). Similar results were obtained for GO annotation and KEGG analysis of SEGs in four development stages (Supplementary Table S6).

**Figure 4:**
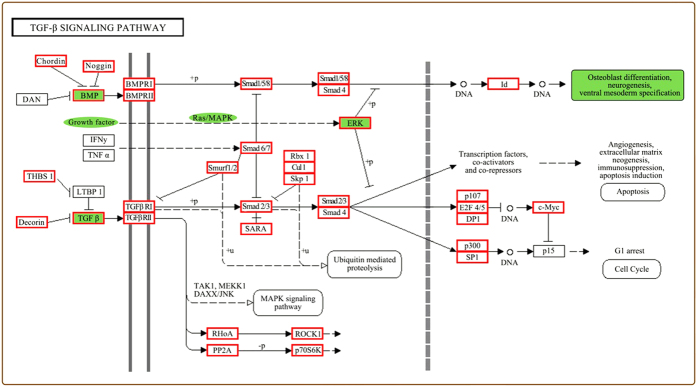
Map of a portion of the TGF-β pathway. Red rectangles represent active gene expression. Green areas represent key signaling pathways and factors involved in the osteoclast differentiation.

In order to narrow the focus of our analyses to genes likely to be relevant to bone tissue development, we performed a detailed analysis of expression levels for genes involved in the TGF-β pathway, which is a known important factor in osteoblast differentiation and bone formation^32^. Twenty-six TGF-β genes were found to show a sustained decline in expression levels, while 14 TGF-β genes showed sustained increases in expression from stages S1 to S4 (Fig. 3C). By referring to the KEGG map of the TGF-β pathway, we found that the TGF-β pathway cooperated with the MAPK and extracellular signal-regulated kinase (ERK) pathways to regulate and control osteoblast differentiation (Fig. 4). It is noteworthy that Unigene34269 and Unigene250 were down-regulated in the ERK 1/2 pathway and Unigene32329 was down-regulated in TGF-β_2_ pathway. Unigene39770 and Unigene20588, which belong to the BMP (bone morphogenetic protein) signaling pathway, were differentially expressed in S1 and S3. Previous studies have reported that ERK plays a significant role in survival of osteoclasts and BMP promote differentiation of meschenchymal cells into chondrocytes and osteoblasts^34^,^35^,^36^,^37^.

### Sequence and expression profiling of IB development-dependent microRNAs

A total of 12,105,829, 11,468,831, 12,420,544 and 12,472,082 raw reads were successively collected from four libraries, respectively. After discarding low-quality reads, 3′ and 5′ adaptors and sequences with <18nt, 11,807,206, 11,222,117, 12,142,432 and 12,042,698 clean small RNA reads were obtained in the S1, S2, S3 and S4 libraries, respectively (Supplementary Table S8-1). Little differences in the length distribution of the sequences from the four libraries was apparent. Most of the small RNAs were 21–23 nt in length, with 22 nt being the most abundant length (>75%) in all libraries. The total number of unique reads from S1, S2, S3 and S4 were 100,606, 78,655, 121,567 and 91,580, respectively. The analysis of common and specific sequences between libraries is shown in Supplementary Table S8-2. Through mapping, 10,432,263 (88.36%), 9,957,281 (88.73%), 10,714,729 (88.24%) and 10,470,015 (86.94%) clean reads representing 20,890 (20.76%), 14,952 (19.01%), 20,108 (21.96%) and 28,282 (23.26%) unique sRNAs were mapped to the reference transcriptome. After NCBI Genebank and Rfam database alignment, rRNA, tRNA, snRNA and snoRNA were annotated and removed (Supplementary Table S8-3). For further miRNA analysis, a total of 420 known miRNAs and 41 novel miRNA candidates were generated. Among them, 375, 360, 358 and 385 known miRNAs and 22, 19, 14 and 23 novel miRNAs were found in the S1, S2, S3 and S4 libraries, respectively (Supplementary Table S9).

**Figure 5:**
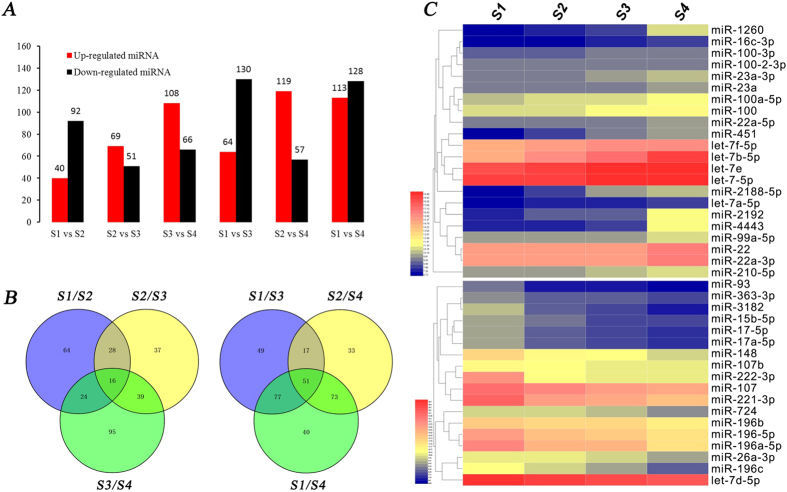
Differentially expressed profiles for miRNAs. (**A**) Number of up/down expressed miRNAs in each comparison analysis group; (**B**) Venn diagram of miRNA differential expression between adjacent/nonadjacent pairwise comparisons; (**C**) Hierarchical cluster analysis of miRNAs, having a sustained decreased/increased expression from stages S1 to S4.

To evaluate the time course and development-dependent miRNA activities across the four development stages of IBs, we performed a time course differential expression miRNA analysis by comparing each two adjacent developmental stages (Supplementary Table S10). Using |log2Ratio| ≥ 1, P < 0.05 and reads ≥ 100 as the cut-off, we identified 132 (40 up-regulated and 92 down-regulated), 120 (69 up-regulated and 51 down-regulated) and 174 (108 up-regulated and 66 down-regulated) differentially expressed miRNAs in adjacent pairwise comparisons of S1-vs-S2, S2-vs-S3 and S3-vs-S4 (Fig. 5A). In nonadjacent pairwise comparisons, 194 (64 up-regulated and 130 down-regulated), 176 (119 up-regulated and 57 down-regulated) and 241 (113 up-regulated and 128 down-regulated) differentially expressed miRNAs were identified in S1-vs-S3, S2-vs-S4 and in S1-vs-S4, respectively (Fig. 5A). Similar to the mRNAs, comparisons of nonadjacent developmental stages obtained more differentially expressed miRNAs (Fig. 5A), and miRNA expression showed a long-term slowly increase/decrease with the IB development. Through Venn diagram analysis, 16 and 51 overlapped differentially expressed miRNAs were identified in the three adjacent pairwise comparisons and three nonadjacent pairwise comparisons, respectively (Fig. 5B). In order to further understand the regulatory functions of miRNAs, a total of 52,918 unique targets of *M. amblycephala* were predicted for the 420 known miRNAs and 41 novel miRNAs including 362 highly significant differently expressed miRNAs.

Furthermore, in order to obtain significant miRNAs, which expression has a sustained increase/decline trend with the development of IBs, a comprehensive analysis was performed using standardized reads data from four stages. Twenty-two miRNAs with sustained increased expression and 18 miRNAs with sustained decreased expression from S1 to S4 stages were detected (Fig. 5C). Functions of these miRNAs in differentiation and development of bone have been verified in many species. For instance, prior studies provided evidence that let-7 miRNAs and miR-140 play major roles in endochondral bone development^38^. Let-7 and miR-22 had also been shown to be positive regulators of bone development, promoting osteogenesis and suppressing adipogenesis of MSCs *in vitro*^39^,^40^. MiR-100, miR-26a and miR-148 play important roles in osteogenic differentiation and osteoclastogenesis, respectively^41^,^42^,^43^. A previous study also found that over-expression of the Hox-cluster miR-196 in zebrafish embryos reduces the number of ribs and somites. Reciprocally, miR-196 knockdown could evoke extra ribs and somites^44^. Therefore, some of the miRNAs that are differentially expressed between these important developmental stages are likely to have important roles in affecting osteoblast differentiation and osteogenesis.

### Analysis of miRNA-mRNA interaction in different developmental stages

The investigation of individual miRNA-target interactions and the miRNA-target interaction network has been an exciting and challenging field of study. In general, increased miRNA activity has been found to reduce the expression of mRNA targets, and vice versa^45^,^46^. Therefore, integrated analysis of miRNA and mRNA expression profiles over the four developmental stages is an effective way to identify functional miRNA-mRNA interaction pairs involved in specific biological processes^47^,^48^.

**Figure 6:**
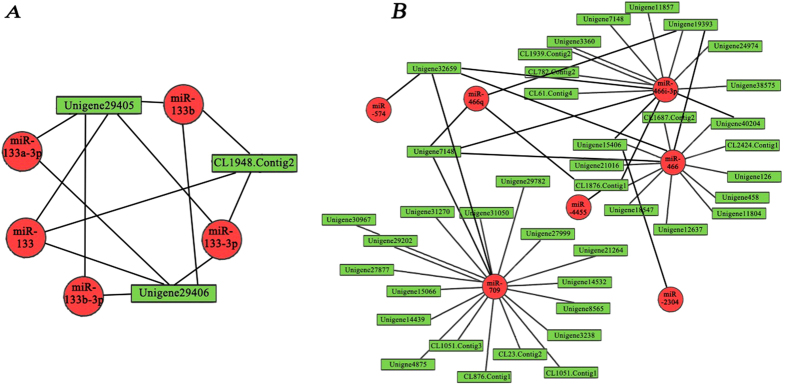
MiRNA-mRNA interaction diagrams. (**A**) MiRNA-mRNA interaction pairs associated with the miR-133 family; (**B**) Visualization of a portion of the miRNA-regulated network.

Through the simultaneous analysis of expression profiles for miRNA and target mRNA involved in IB development, we identified 201, 31, 92, 219, 93 and 500 miRNA-mRNA interaction pairs from six pairwise comparisons (S1-vs-S2, S2-vs-S3, S3-vs-S4, S1-vs-S3, S2-vs-S4 and S1-vs-S4), respectively (Supplementary Table S11). For the miRNA-mRNA interaction pairs, we found that some miRNAs were closely related to bone formation and differentiation, such as miR-133/133a/133b, miR-222 and miR-3960^49^,^50^,^51^. The interaction map of our study performed that CL1948.Contig2 (S1-S4) was down-regulated with the up-regulated of miR-133, miR-133-3p and miR-133b, while unigene29405 and unigene29406 interact with miR-133, miR-133-3p and miR-133b (Fig. 6A). This suggests that these miRNA-mRNA interaction pairs may function in IB formation. Furthermore, in order to detect the functional characteristics of miRNA-mRNA interaction pairs, the mRNAs involved in interaction pairs were subjected to a GO analysis and KEGG functional annotation (Supplementary Table S11). The mRNAs identified in these pairs included those involved in the TGF-beta signaling pathway, Osteoclast differentiation, MAPK signaling pathway, Wnt signaling pathway and Calcium signaling pathway (Supplementary Table S11). For instance, CL712.Contig1 (S1–S2, S2–S3, S1–S3), Unigene28219 (S3–S4), Unigene2429 (S1–S3), Unigene20803 (S2–S4, S1–S4) and Unigene6271 (S1–S4) were mapped to the TGF-β signaling pathway; Unigene7819 (S1–S2, S1–S3, S1–S4), Unigene41990 (S3–S4, S2–S4, S1–S4), Unigene271 (S3–S4, S1–S4) and CL782.Contig2 (S1–S3, S1–S4) were simultaneously mapped to the Osteoclast differentiation and MAPK signaling pathway. Similarly, other genes like Unigene34104 (S1–S2, S1–S3), Unigene25273 (S2–S3) and CL618.Contig10 (S1–S4) were mapped to the Calcium signaling pathway and Unigene31459 (S3–S4, S2–S4, S1–S4) was mapped to the Wnt signaling pathway. Previous research has suggested that TGF-β and Wnt are involved in many cellular processes affecting osteoblast differentiation and bone formation^32^,^52^,^53^,^54^. Another pathway targeted by the mRNAs was the MAPK signaling pathway, which is not only known to be a major regulator of skeletal muscle development^55^,^56^, but is also essential for bone development and maintenance^57^. In addition, Ca^2+^ signals play key roles in osteoblast function^58^,^59^. Collectively, the existing literature supports that many of the miRNA-mRNA interaction pairs identified in our study are likely to be involved in regulating development and differentiation of bone.

### Quantitative analysis of miRNA and mRNA expression

**Figure 7:**
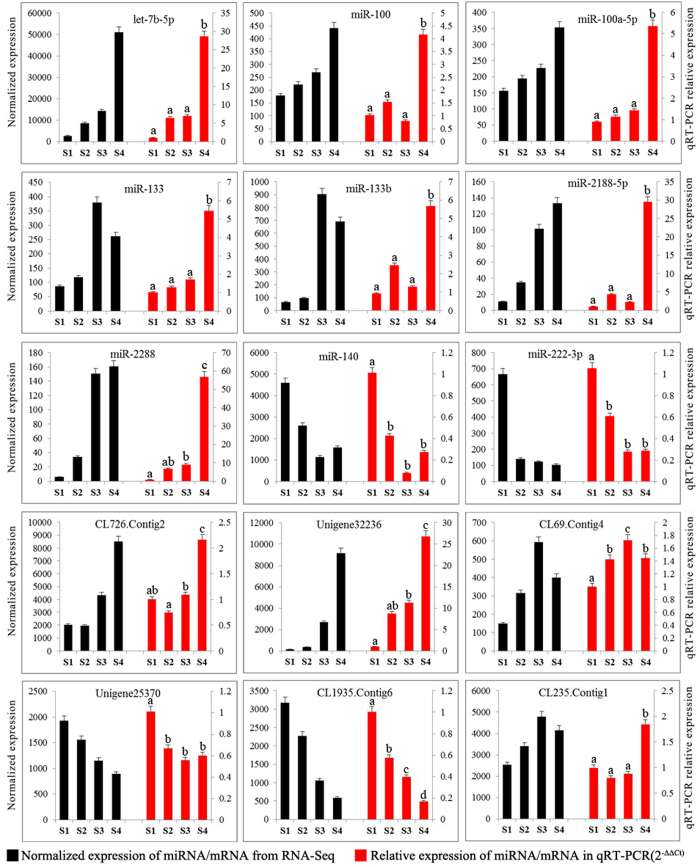
Comparison of expression levels for the 9 detected miRNAs and 6 mRNAs using RNA-Seq and qRT-PCR. All the data were shown as mean ± SE. The different letters for qRT-PCR results mean significant difference between stages (P < 0.05).

**Figure 8:**
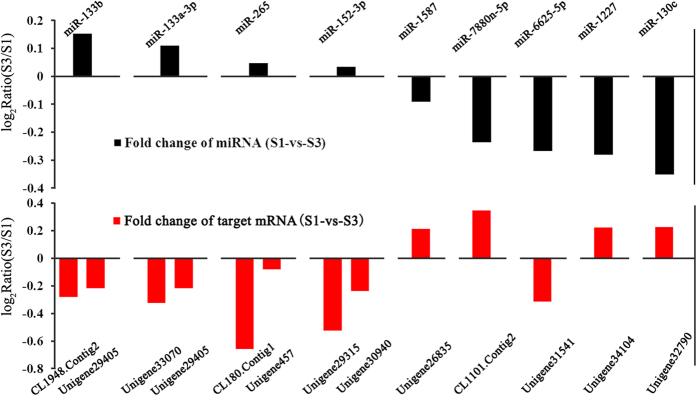
Verification of expression patterns of miRNA-mRNA interaction pairs using qRT-PCR.

Quantitative PCR results for 9 miRNAs and 6 mRNAs revealed similar changes in expression to that detected using RNA-seq data (Fig. 7). A similar result was also observed in the validation of the miRNA-mRNA interaction pairs, and 12 of 13 interaction pairs have a same expression pattern with the sequencing data except one of them performed a down-down regulating pattern (Fig. 8). We think the result is acceptable and it confirmed the credibility of molecular resources and sequencing data identified in our study. Because extensive researches performed that hundreds of miRNAs interact with thousands of target mRNAs to maintain proper gene expression patterns under various physiological functions, as the results we showed. The complexity of the miRNA-mRNA interaction network presents a great challenge for researchers to reveal the roles for specific miRNAs or miRNA-mRNA interactions in specific biological processes^60^. For instance, miR-709, miR-466 and miR-466i-3p constituted a complex interaction network with unigene32659, unigene7148, unigene15406 and many other miRNAs and mRNAs (Fig. 6B). Furthermore, miRNA-mRNA interaction is only one of the multiple mechanisms influencing the regulation of gene expression and our results will not be sensitive in instances when multiple factors, in addition to miRNA-mRNA interactions are involved^61^,^62^. Moreover, in order to explore IB specific genes, we performed expression analysis of 65 bone-related genes in IBs, muscle and rib tissues. However, the present results indicated that it was not possible, despite repeated attempts to demonstrate IB specific localization of gene from the transcriptome. The failure to demonstrate IB specific gene expression may be because of the fact that the transcriptome contained genes not only for IB, particularly since genes inducing IB development may be expressed in associated tissues, such as muscle. Clearly further research will be required to confirm the function of genes involved in IB development.

### Target verification of miR-133b-3p and miR-206-3p

**Figure 9:**
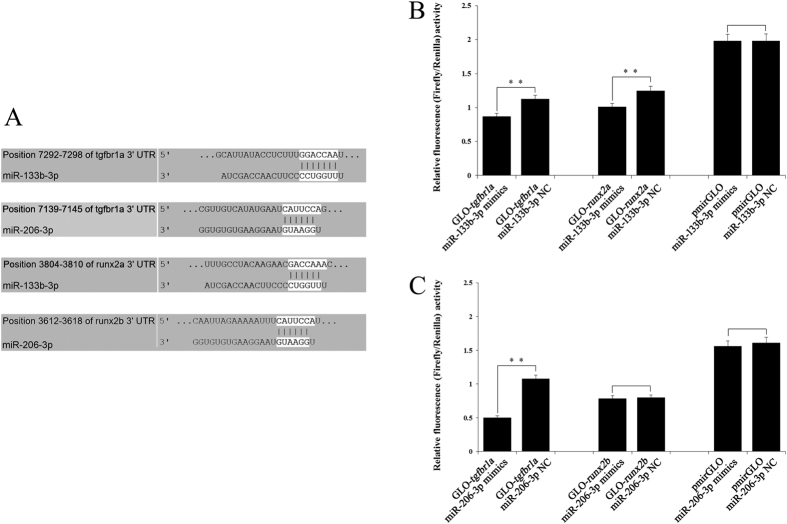
Target verification of miR-133b-3p and miR-206-3p. (**A**) MiRNA seed sequences and their complementary sequences on 3′ UTR; (**B**) miR-133b-3p mimics were cotransfected with *tgfbr1a* or *runx2a* 3′ UTR dual-luciferase reporters into HeLa cells; (**C**) miR-206-3p mimics were cotransfected with *tgfbr1a* or *runx2b* 3′ UTR dual-luciferase reporters into HeLa cells. Firefly luciferase activities were detected and normalized to renilla luciferase (internal control). Results represent means ± SE (n = 4, **P < 0.01).

Previous research supports that the miR-133 family and miR-206 were involved in bone formation and differentiation^63^. *Tgfbr1a* and *runx2* genes, which functioned in skeletal development^32^,^64^, were identified as downstream targets of the miR-133b-3p/miR-206-3p cluster, with complementary binding sites detected on the 3′ UTRs (Fig. 9A). To evaluate the functions of miR-133b-3p/miR-206-3p at target 3′ UTRs, dual-luciferase reporter constructs carrying *tgfbr1a* or *runx2* 3′ UTR (wild-type or empty) were cotransfected with miR-133b-3p mimics or miR-206-3p mimics. The results showed that luciferase activity for the wild-type construct (GLO-*tgfbr1a*, GLO-*runx2a*) was significantly reduced by miR-133b-3p mimics and miR-206-3p mimics, but not by that of the empty construct (pmirGLO) (Fig. 9B,C). The wild-type construct GLO-*runx2b* was not reduced by miR-206-3p mimics (Fig. 9C), which showed the inaccuracy of software prediction and the necessity of experimental verification.

## Conclusion

This is the first integrated analysis of miRNA and mRNA related to IBs development in fish species. MiRNA and mRNA expression profiles involved in IB development were revealed over four developmental stages. Putative miRNAs/genes associated with IB development were identified in the study. In addition, we found that a number of miRNA-mRNA interaction pairs were associated with bone formation and differentiation, adding to knowledge about the regulation of IB development. This study generated fundamental molecular resources for four developmental stages from emerging to complete formation of IBs, which can be used to improve understanding about the molecular processes affecting IB development.

## Additional Information

**How to cite this article**: Wan, S.-M. *et al*. Dynamic mRNA and miRNA expression analysis in response to intermuscular bone development of blunt snout bream (*Megalobrama amblycephala*). *Sci. Rep.* **6**, 31050; doi: 10.1038/srep31050 (2016).

## Supplementary Material

Supplementary Information

Supplementary Data

## Figures and Tables

**Table 1:** Assembly and annotation results of transcriptome sequencing.

Category	Number	Database	Number of unigenes
Total Raw Reads	92,991,884	NR	33,354
Total Clean Reads	87,582,724	NT	45,648
Q20 percentage	98.11%	Swiss-Prot	29,753
GC percentage	48.70%	KEGG	24,094
Total number of contigs	127,712	COG	9,072
Total length (bp) of contigs	38,443,923	GO	22,222
Mean Length (bp) of contigs	301	Total Number of annotated unigenes	46,569
N50 of contigs	437		
Total number of unigenes	52,918		
Total length (bp) of unigenes	33,582,035	None annotated unigenes	6,349
Mean length (bp) of unigenes	635		
N50 of unigenes	865		

**Table 2:** Alignment results of 8 libraries mapping to the reference transcriptome.

Sample ID	Total reads	Total base pairs	Total mapped reads	Unique match	Multi-position match	Total unmapped reads
StageI-1	14,010,101 (100.00%)	1,893,716,998 (100.00%)	13,518,462 (96.49%)	8,802,813 (62.83%)	4,715,649 (33.66%)	491,639 (3.51%)
StageI-2	15,412,123 (100.00%)	2,149,413,540 (100.00%)	14,947,070 (96.98%)	9,564,804 (62.06%)	5,382,266 (34.92%)	465,053 (3.02%)
StageII-1	12,371,018 (100.00%)	1,739,928,464 (100.00%)	12,028,229 (97.23%)	7,172,029 (57.97%)	4,856,200 (39.25%)	342,789 (2.77%)
StageII-2	15,891,229 (100.00%)	2,332,924,677 (100.00%)	15,533,624 (97.75%)	9,456,435 (59.51%)	6,077,189 (38.24%)	357,605 (2.25%)
StageIII-1	15,194,682 (100.00%)	2,104,759,656 (100.00%)	14,838,649 (97.66%)	8,411,453 (55.36%)	6,427,196 (42.30%)	356,033 (2.34%)
StageIII-2	12,908,078 (100.00%)	1,758,036,319 (100.00%)	12,612,680 (97.71%)	6,935,639 (53.73%)	5,677,041 (43.98%)	295,398 (2.29%)
StageIV-1	16,179,709 (100.00%)	2,200,713,831 (100.00%)	15,877,084 (98.13%)	9,491,808 (58.66%)	6,385,276 (39.46%)	302,625 (1.87%)
StageIV-2	18,600,312 (100.00%)	2,583,939,574 (100.00%)	18,324,379 (98.52%)	10,852,126 (58.34%)	7,472,253 (40.17%)	275,933 (1.48%)
