# Supplementary material for: Dynamic mRNA and miRNA expression analysis in response to intermuscular bone development of blunt snout bream (Megalobrama amblycephala)
Source: Sci Rep. 2016 Aug 3;6:31050. doi: 10.1038/srep31050 (PMC4971466; doi:10.1038/srep31050)
Supplement: Supplementary Information [file srep31050-s1.doc]

**Dynamic mRNA and miRNA expression analysis in response to intermuscular bone development of blunt snout bream (*Megalobrama amblycephala*)**

Shi-Ming Wan^1,2^, Shao-Kui Yi^1,2^, Jia Zhong^1,2^, Chun-Hong Nie^1,2^, Ning-Nan Guan^1,2^, Wei-Zhuo Zhang^1,2^, Ze-Xia Gao^1,2,3*^

^1^ College of Fisheries, Key Lab of Agricultural Animal Genetics, Breeding and Reproduction of Ministry of Education/Key Lab of Freshwater Animal Breeding, Ministry of Agriculture, Huazhong Agricultural University, Wuhan, Hubei 430070, China

^2^ Freshwater Aquaculture Collaborative Innovation Center of Hubei Province, Wuhan 430070, China

^3^ Hubei Provincial Engineering Laboratory for Pond Aquaculture, Wuhan 430070, China

*Corresponding author: Ze-Xia Gao, College of Fisheries, Huazhong Agricultural University, Wuhan, 430070 Hubei, China. E-mail address: gaozexia@hotmail.com

**Keywords:** intermuscular bone; development; RNA-seq; miRNA-mRNA interaction; blunt snout bream


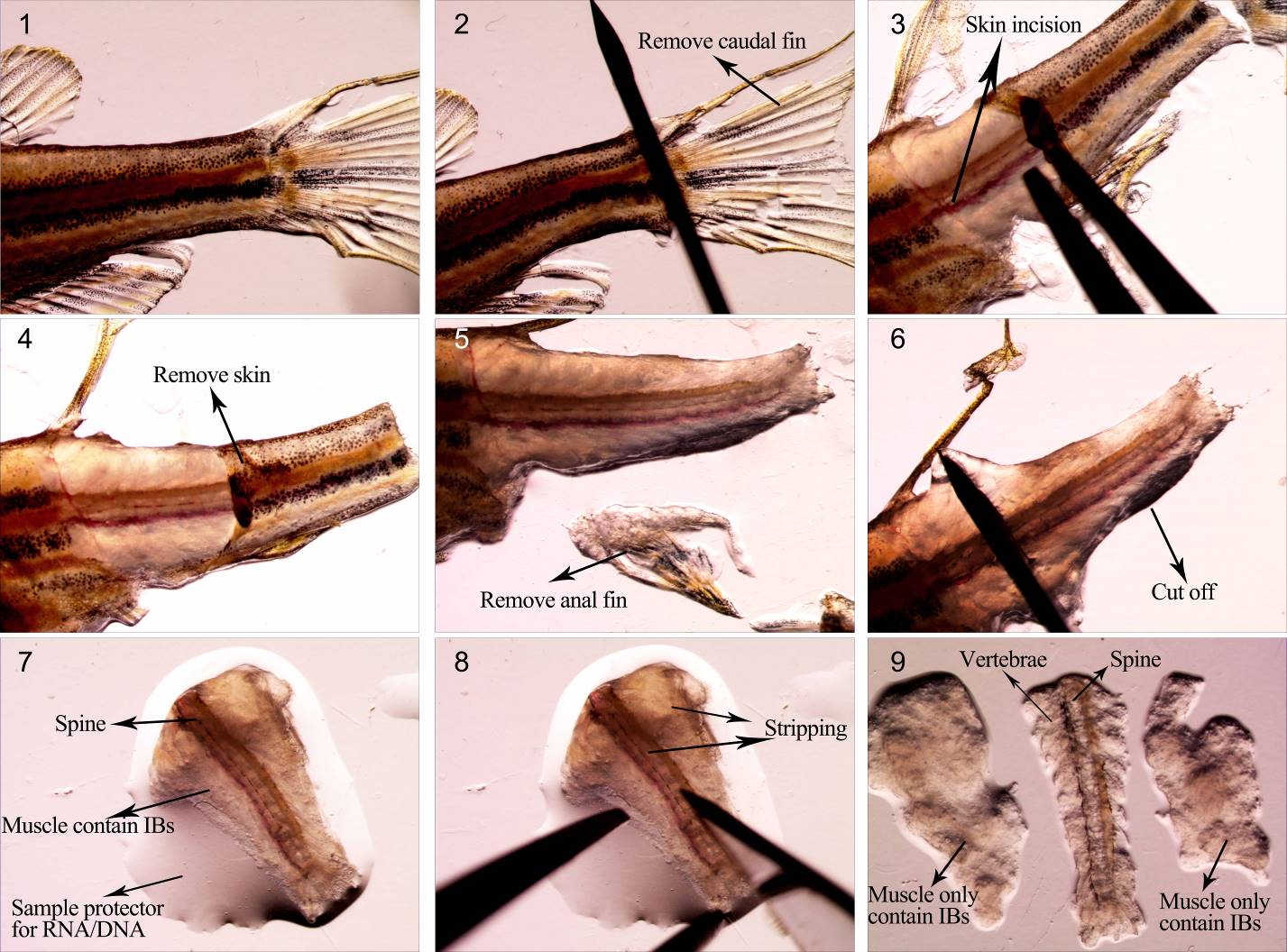


**Figure S1-1** Sampling procedure of muscle tissue contained IBs


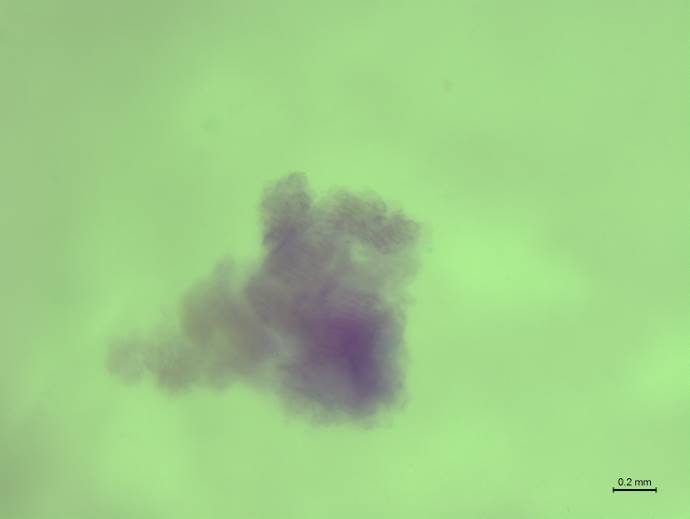

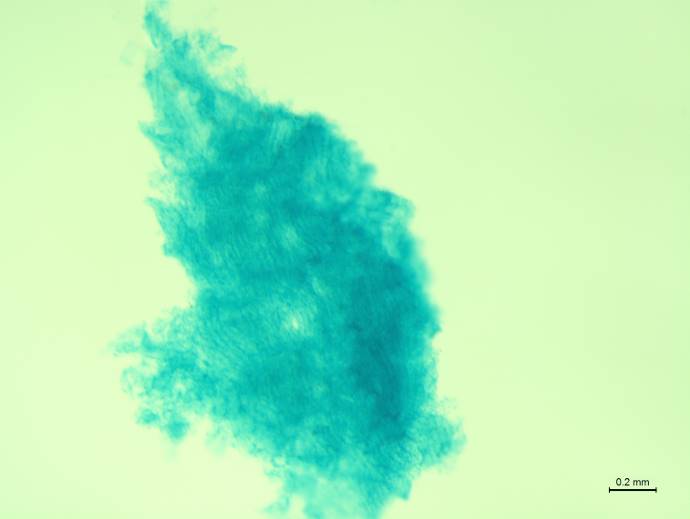
 **Alizarin red staining, Stage I Alcian blue staining, Stage I**


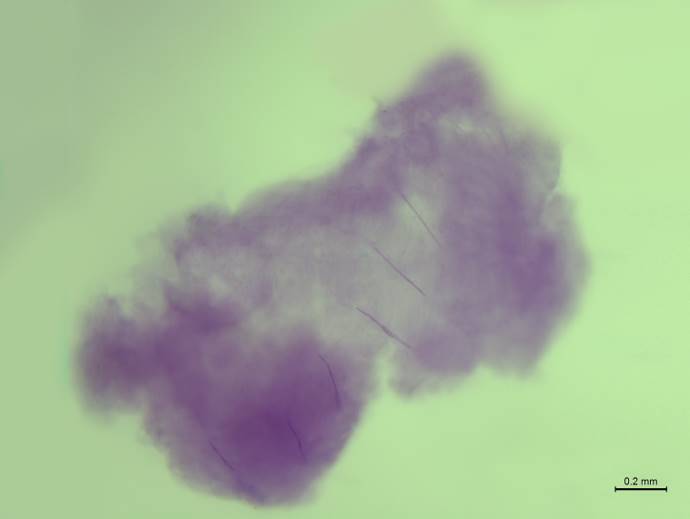

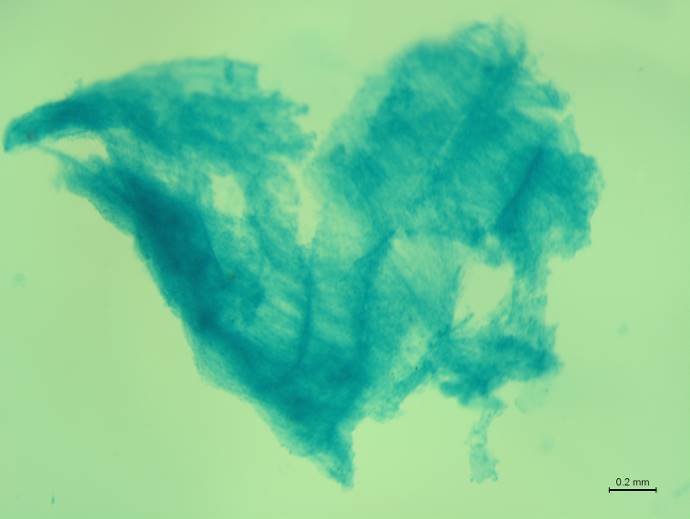


**Alizarin red staining, Stage II Alcian blue staining, StageII**


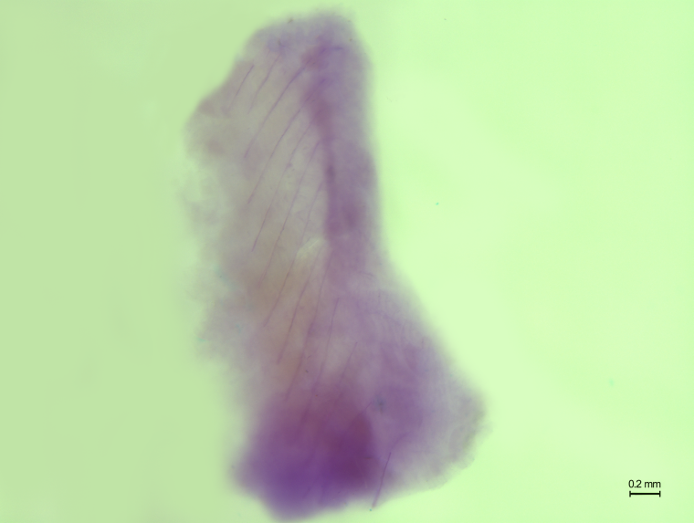

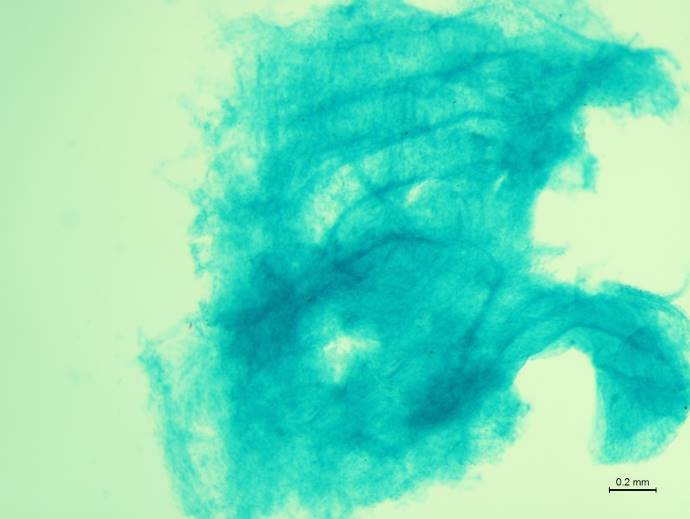


**Alizarin red staining, Stage III Alcian blue staining, Stage III**


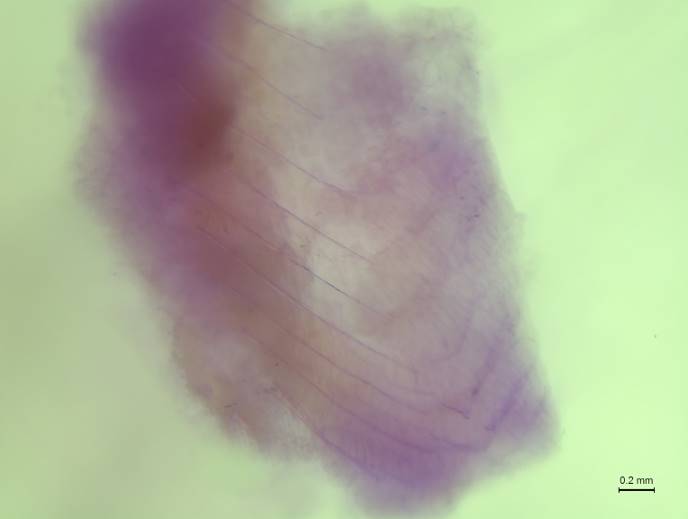

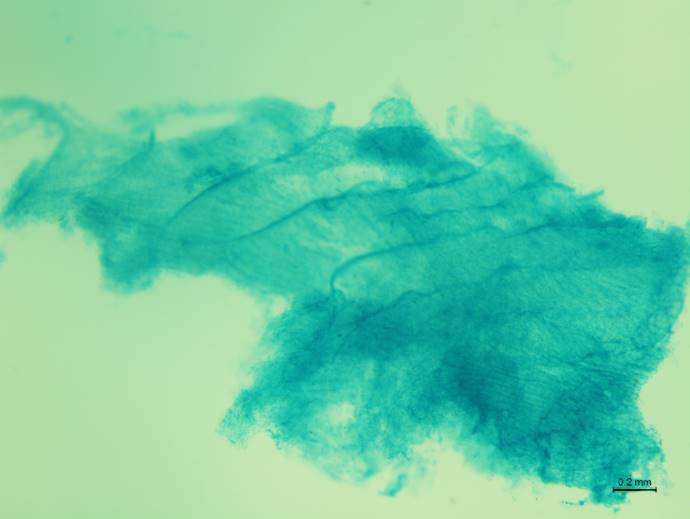


**Alizarin red staining, Stage IV Alcian blue staining, Stage IV**


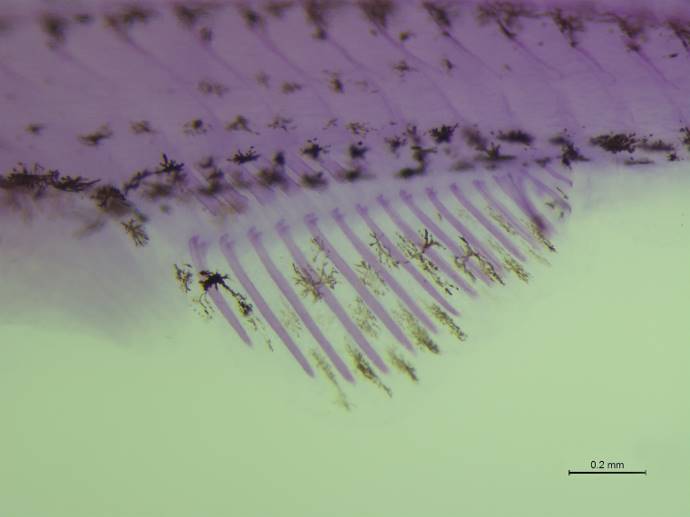

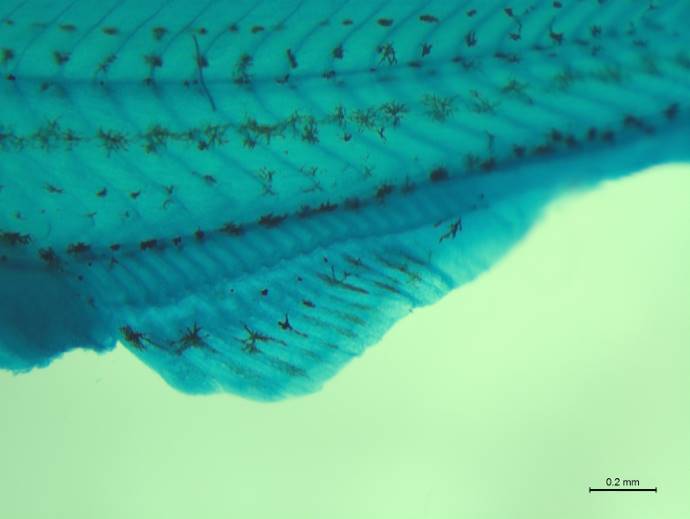


**Alizarin red staining, Anal fin Alcian blue staining, Anal fin**


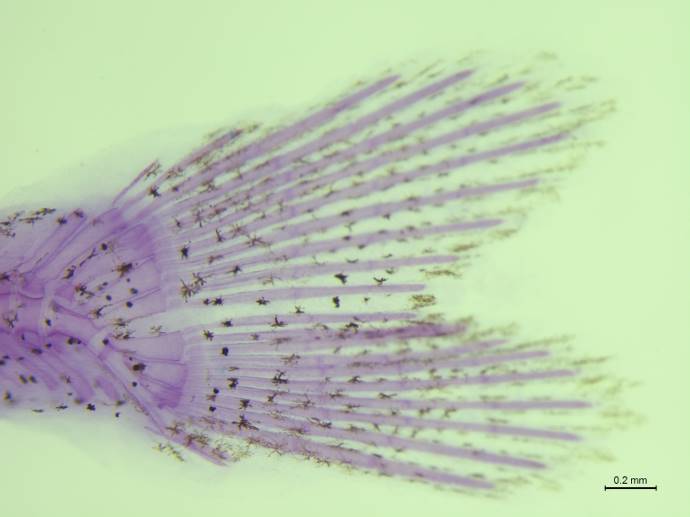

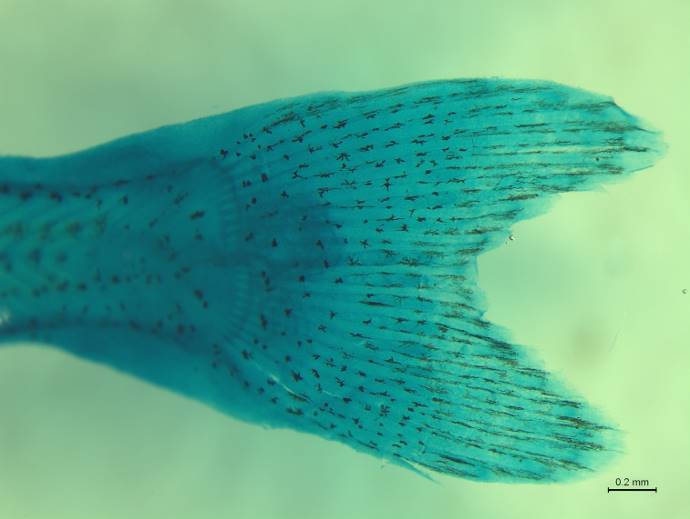


**Alizarin red staining, Caudal fin Alcian blue staining, Caudal fin**

**Figure S1-2** Tissue sample stained with Alcian blue and Alizarin red

Alizarin red staining tissues are showed on the left side of the figure and Alcian blue staining tissues are shown on the right side of the figure. The results clearly indicate that the tissue samples used for sequencing contained only muscle and intermuscular bone without any other irrelevant tissues.


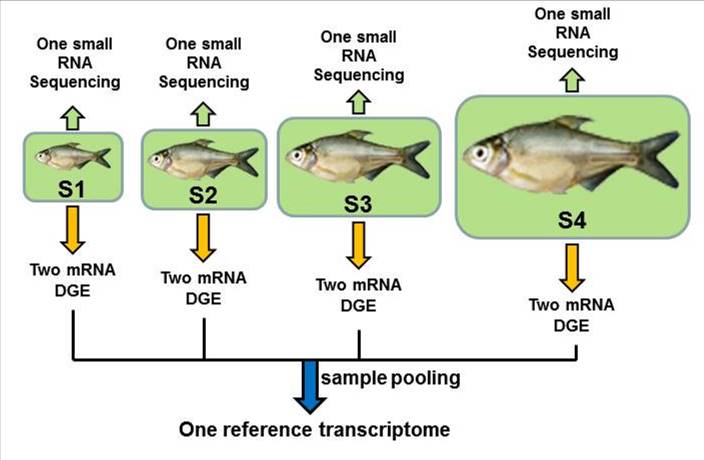


**Figure S1-3** A visual map of library construction


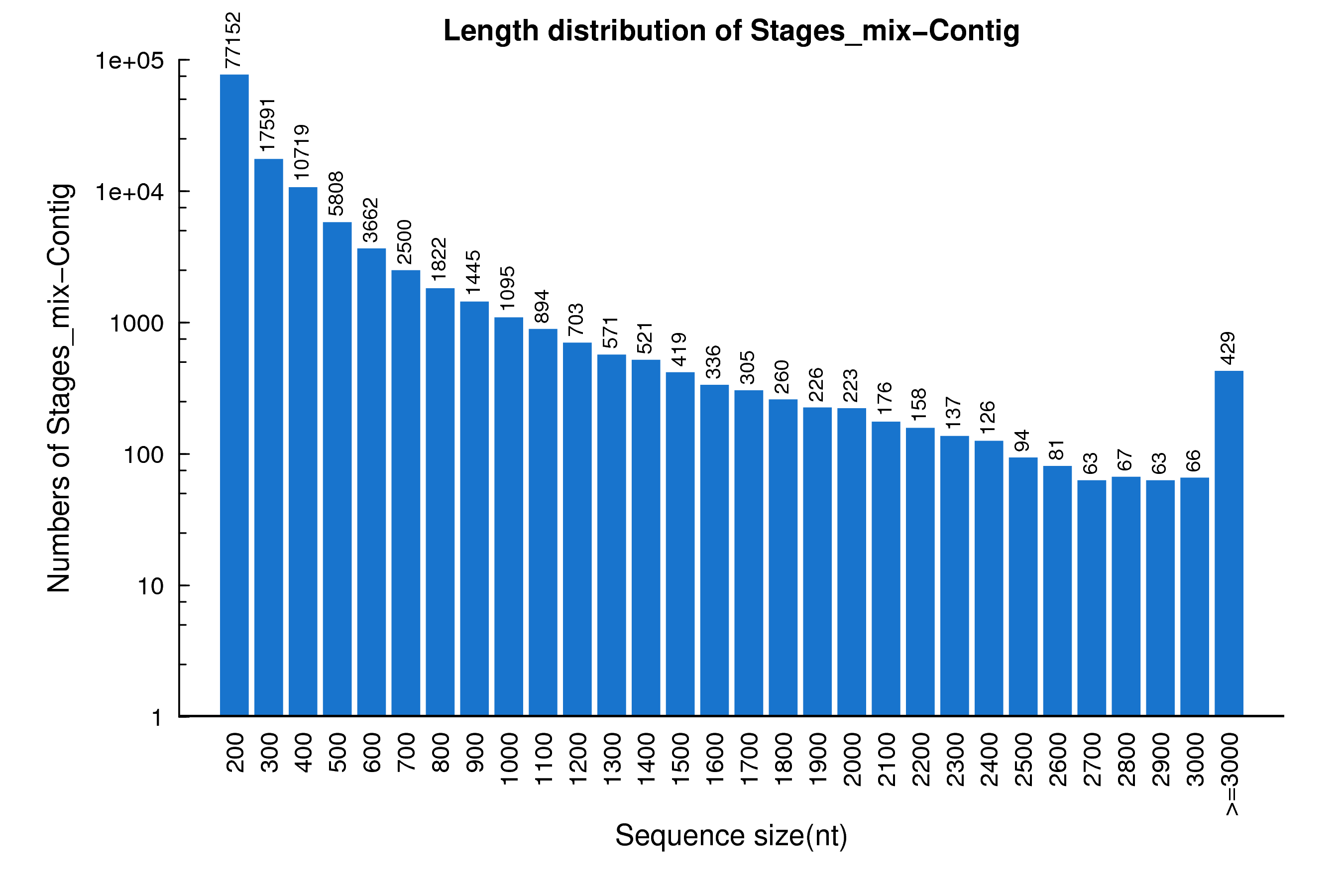


**Figure S1-4** Length distribution of contigs in the transcriptome


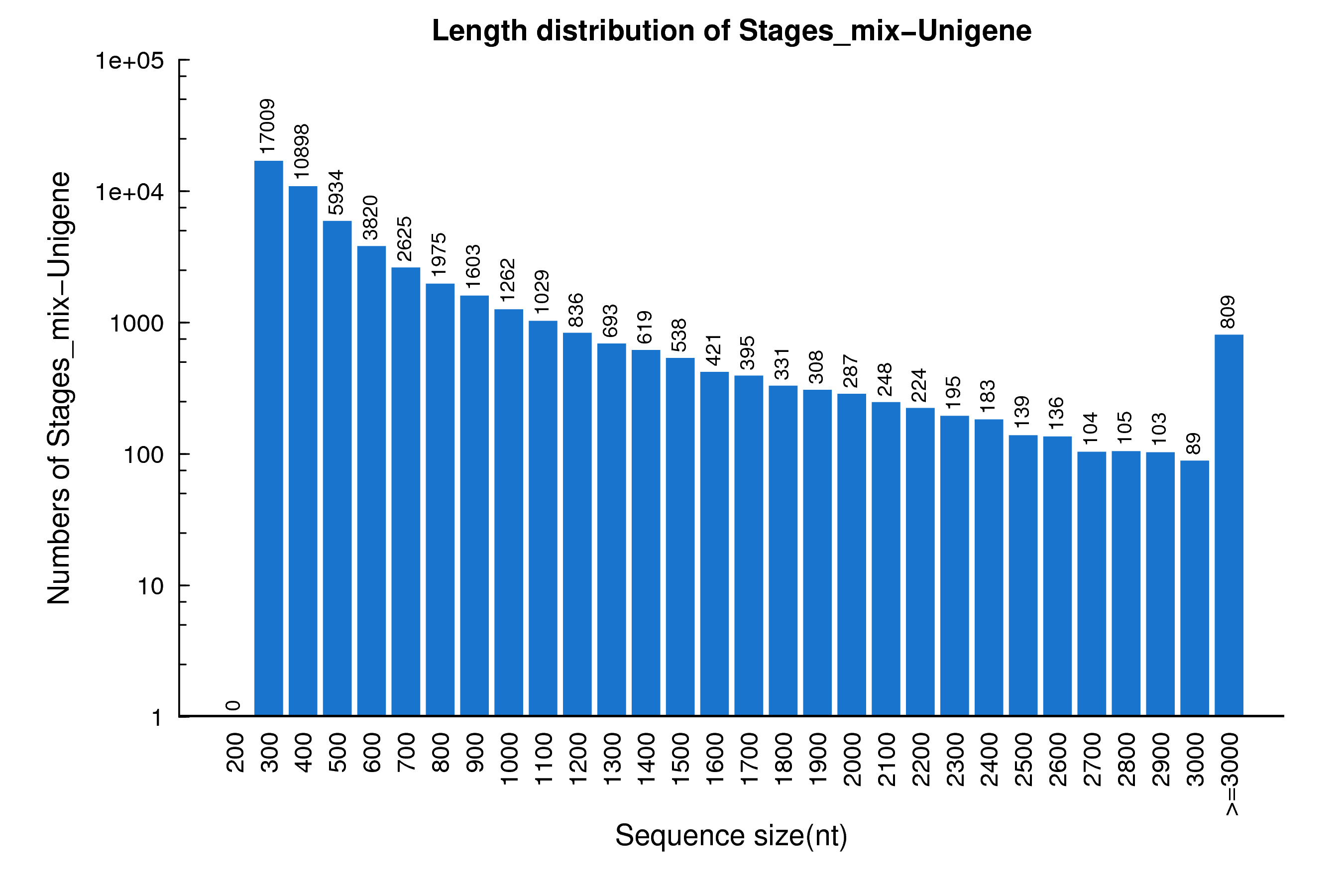


**Figure S1-5** Length distribution of unigenes in the transcriptome


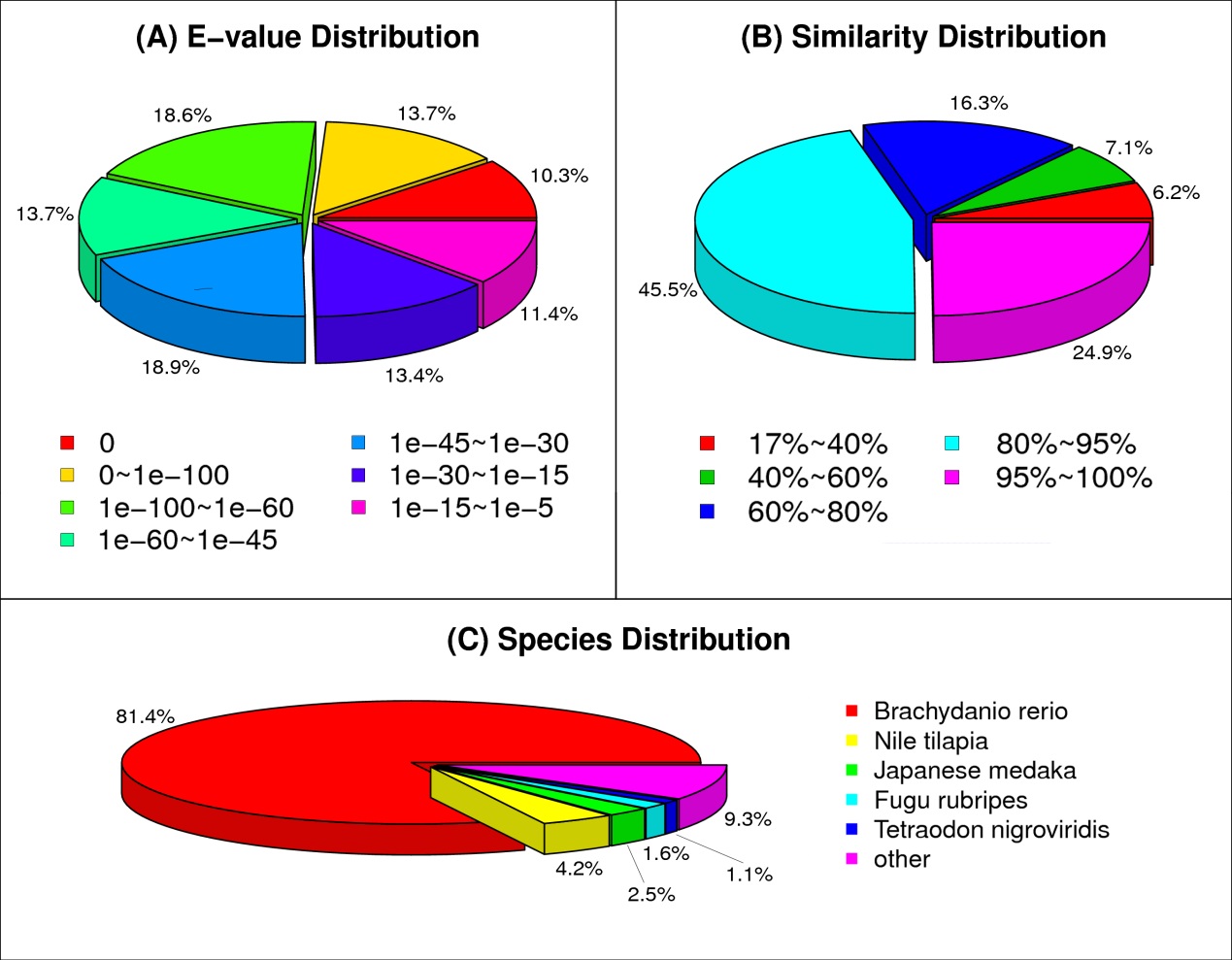


**Figure S1-6** The results of NR annotation

(A): The E-value distribution of the result of NR annotation. (B): The similarity distribution of the result of NR annotation. (C): The species distribution of the result of NR annotation.


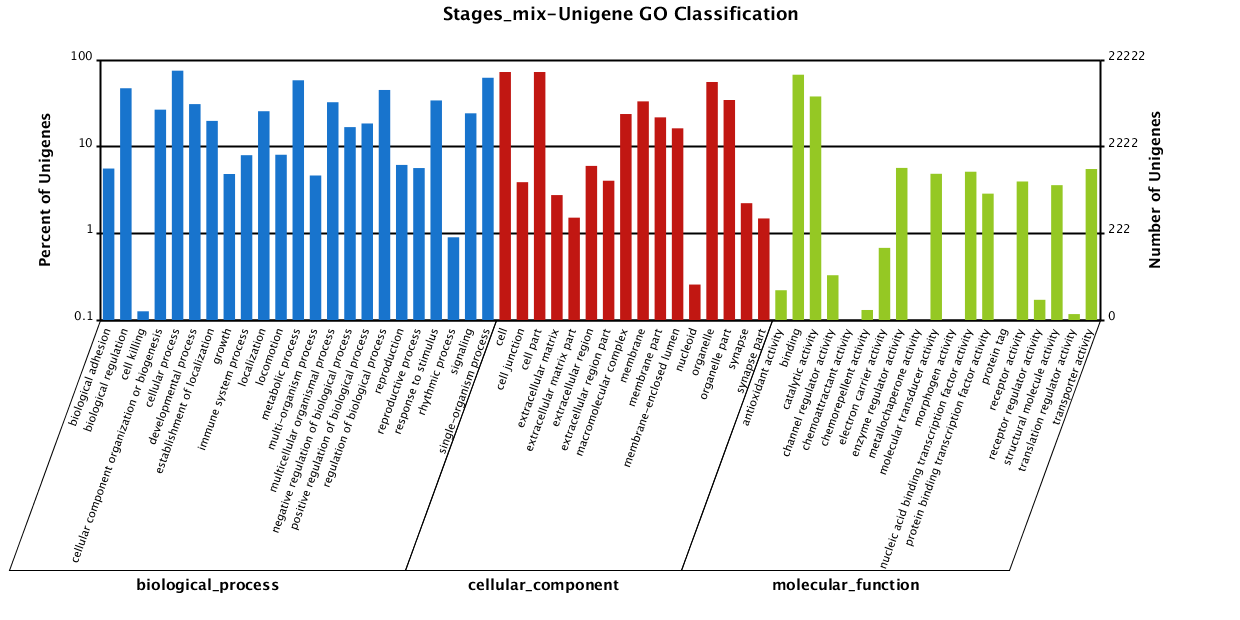
**Figure S1-7** GO classification analysis of Unigenes

GO functions is showed in X-axis. The right Y-axis shows the number of genes which have the GO function, and the left Y-axis shows the percentage.


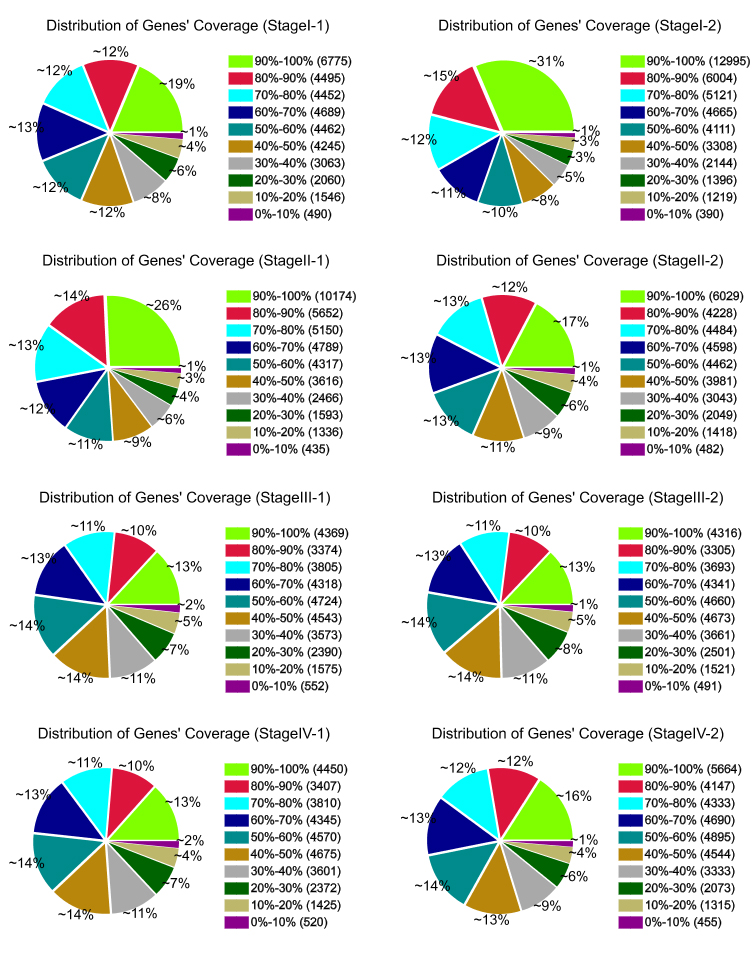


**Figure S1-8** Gene coverage of 8 DGE libraries

Gene coverage is the percentage of a gene covered by reads. This value is determined as the ratio of the base number in a gene covered by unique mapping reads to the total bases number of that gene.


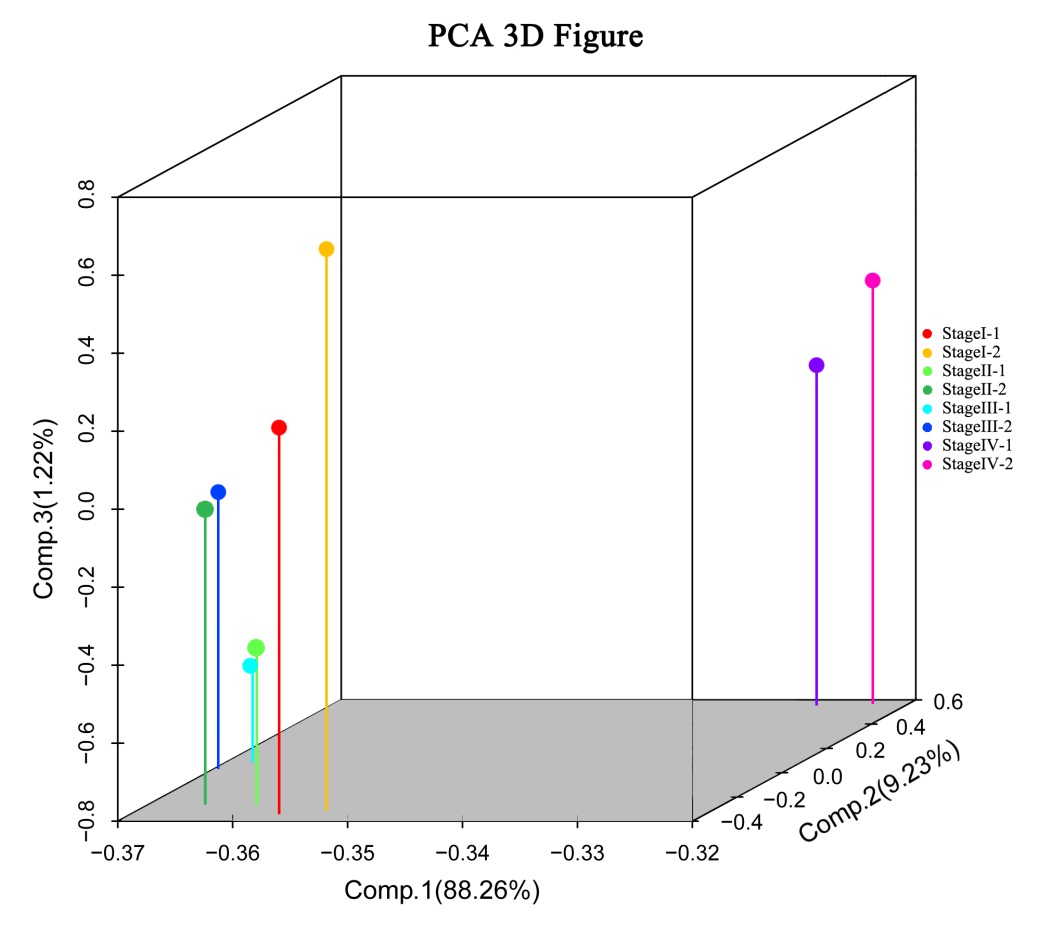


**Figure S1-9** Principal component analysis (PCA) of 8 DGE libraries in the 4 stages

The lines of different colors represent different DGE libraries. The length of the lines and the distance between lines represents the difference between each other.
